# Ten-year experiences with Tetanus at a Tertiary hospital in Northwestern Tanzania: A retrospective review of 102 cases

**DOI:** 10.1186/1749-7922-6-20

**Published:** 2011-07-08

**Authors:** Phillipo L Chalya, Joseph B Mabula, Ramesh M Dass, Nkinda Mbelenge, Stephen E Mshana, Japhet M Gilyoma

**Affiliations:** 1 Department of Surgery, Weill-Bugando University College of Health Sciences, Mwanza, Tanzania; 2 Department of Orthopedics, Weill-Bugando University College of Health Sciences, Mwanza, Tanzania; 3 Department of Microbiology, Weill-Bugando University College of Health Sciences, Mwanza, Tanzania

**Keywords:** Tetanus, clinical characteristics, treatment outcome, predictors of outcome, Tanzania

## Abstract

**Background:**

Tetanus is still a major health problem in developing countries and it is associated with a high morbidity and mortality rate. There is paucity of published data regarding the management of tetanus in Tanzania, especially the study area. This study was conducted to describe our own experiences with tetanus outlining the clinical characteristics and treatment outcome of tetanus patients in our environment and to identify predictors of outcome of these patients.

**Methods:**

This was a ten-year period retrospective study of patients who presented with a clinical diagnosis of tetanus at Bugando Medical Centre between January 2001 and December 2010. Data was analyzed using SPSS computer software system.

**Results:**

A total of 102 patients were studied. The male to female ratio was 11.8: 1. The majority of patients (74.5%) were aged < 40 years and 51.0% of them were farmers. Only 23.5% of patients had prior tetanus immunization. 53.5% of patients had a reasonably identifiable acute injury prior to the onset of tetanus and commonly involved the lower limbs (53.8%). The majority of patients (97.1%) had generalized tetanus. The mean incubation period and period of onset were 8.62 ± 4.34 and 3.8 ± 2.2 days respectively. Complication rate was 54.9%. The average overall duration of hospitalization was 34.12 ± 38.44 days (1-120 days). Mortality rate was 43.1%. According to multivariate logistic regression analysis, the age ≥ 40 years (P = 0.002), incubation period < 7 days (P = 0.014), tracheostomy (P = 0.004), severity of tetanus (P = 0.001) and need for ventilatory support (P = 0.013) were found to be significantly associated with higher mortality.

**Conclusion:**

Tetanus remains a major public health problem in our centre and still carries unacceptably high morbidity and mortality despite the available advanced management facilities including ICU care. Young adult males are commonly affected. The incidence of tetanus can be reduced significantly by an effective immunization program and proper wound management of the patients. Early recognition, intense support and prompt treatment improves morbidity and mortality of patients diagnosed with tetanus.

## Background

Tetanus, though a vaccine preventable disease, is still a significant public health problem throughout the world and it is associated with a high morbidity and mortality rate, particularly in the developing world [1-3]. The global incidence of tetanus is still estimated at one million cases annually, with a case fatality ratio ranging from 6% to 72% depending on the availability of well equipped intensive care unit [3]. The incidence of tetanus in the developed world is markedly low and is no longer responsible for significant mortality, this has been attributed to high level of health awareness in terms of vaccination and availability of human and material resources to manage the disease [4]. In developed countries tetanus occurs mainly in elderly due to decline in protective antibodies [5,6] and in developing countries tetanus is common in the young due to lack of effective immunization program and appropriate treatment of injuries [4,7].

Tetanus is caused by *Clostridium Tetani*, a gram positive, anaerobic and spore forming bacterium which is found in soil and in animal and human faeces and the usual mode of entry is through a punctured wounds or lacerations, although tetanus may follow surgery, burns, gangrene, chronic ulcers, dog bites, injections such as with drug users, dental infection, abortion and childbirth [3,8]. In some patients no portal of entry for the organism can be identified [5,8].

Tetanus occurs sporadically and almost always affects non-immunized, partially immunized, or fully immunized persons who fail to maintain adequate immunity with booster doses of vaccine [9,10]. It is therefore very important, in order to have protection against tetanus, that all age groups have the universal primary immunization with subsequent maintenance of adequate antitoxin levels by means of appropriately timed boosters [4,10].

The clinical manifestations of tetanus which include trismus (lockjaw), dysphagia, neck stiffness and generalized muscular rigidity is due to a powerful neurotoxin (Tetanospasmin) elaborated by the causative bacterium [11]. Four clinical forms of tetanus are recognized and they include generalized, localized, cephalic and neonatal tetanus [9-11]. Spasm related respiratory compromise, hospital acquired pneumonia and autonomic instability are usually the main causes of morbidity and mortality of this disease [11,12]. The diagnosis of tetanus is most frequently made on clinical manifestations, rather than on bacteriologic findings [8-13].

Management of tetanus patients is too demanding, prolonged, and expensive both in terms of materials and manpower [4,14]. A way to alleviate these problems is by adopting a rigorous tetanus immunization discipline in our community.

In Tanzania, like in most developing countries in the world, tetanus is endemic and remains an important health problem especially among the rural farming folks [4]. Tetanus is one of the most common causes of intensive care unit (ICU) admissions at Bugando Medical Centre (a tertiary hospital in Northwestern Tanzania} and is associated with high morbidity and mortality. This study was undertaken to describe the clinical characteristics and treatment outcome of tetanus patients in our environment and to identify predictors of outcome among these patients.

## Methods

### Study setting and design

This was a ten-year period retrospective study of patients who presented with tetanus at Bugando Medical Centre between January 2001 and December 2010. Bugando Medical Centre {BMC) is tertiary and teaching hospital for the Weill-Bugando University College of Health Sciences (WBUCHS) and is found in Mwanza city along the shore of Lake Victoria in northwestern Tanzania. It is a 1000-bedded hospital and serves approximately 13 million people from its neighboring regions namely Mwanza, Mara, Kagera, Shinyanga, Kigoma and Tabora. The hospital has a 12-bed adult and 10-bed paediatric multi-disciplinary Intensive Care Unit (ICU) which is headed by a consultant anesthesiologist and run by trained ICU nurses. Facilities in the unit include multi-parameter patient monitors, 1 defibrillator, syringe pumps, 2 mechanical ventilators and a standby anesthetic machine for emergency resuscitation when required. Oxygen supply is from oxygen concentrators available in the unit and cylinders provided on request from an oxygen bank in the hospital. The unit has one computer for accurate record keeping and documentation. Tetanus patients are usually admitted in the ICU and treated as per standard protocol for the management of tetanus.

### Study subjects

The study included all patients of all age groups and gender who presented with a clinical diagnosis of tetanus. Patients who had incomplete or missed basic information were excluded from the study. The diagnosis of tetanus was wholly clinical and based on the presence of one or more of the following:-

1. Trismus

2. Rigidity of the neck and or abdomen

3. Reflex spasms

Tetanus was classified into generalized, cephalic, localized and neonatal types. Patients with rigidity and/or spasm limited to the wound bearing area of the body were classified as having localized tetanus, whereas those with trismus and generalized rigidity with or without generalized spasms were classified as having generalized tetanus. Tetanus occurring during neonatal period was classified as neonatal tetanus. A form of localized tetanus restricted to head and neck was classified as cephalic tetanus. The severity of tetanus was classified into mild, moderate severe and very severe according to the system reported by Ablett [15]. The treatment was started immediately once the diagnosis was made. The three objectives of therapy i.e. supportive care; neutralization of circulating toxin and removal (eradication) of the source of tetanospasmin (infected sites) was applied to all cases depending on the severity of spasms and availability of all essential facilities. The patients were treated as per standard protocol for the management of tetanus which included antibiotics (i.e. Penicillin, metranidazole or combination of both), wound care, passive immunization with human tetanus immune globulins (500 Units I/M stat) and active immunization with injection Tetanus Toxiod at the time of admission which was repeated when patient were discharged from the ward. The patients also received Diazepam for the control of spasm and mechanical ventilation when and where it was required. Details of demographic data (i.e. age, sex, occupation), tetanus immunization history, suspected portal of entry of infection, incubation time, clinical presentations, management, related complications, duration of intensive care unit admission, length of hospitalization, outcome and factors predicting the outcome were obtained from medical records and entered in a questionnaire before analysis. Incubation period was defined as the time from injury to the appearance of symptoms and the period of onset was defined as the interval between the first symptoms and the first spasm.

### Statistical analysis

The statistical analysis was performed using statistical package for social sciences (SPSS) version 15.0 for Windows (SPSS, Chicago IL, U.S.A). The mean ± standard deviation (SD), median and ranges were calculated for continuous variables whereas proportions and frequency tables were used to summarize categorical variables. Continuous variables were categorized. Chi-square (χ2) test were used to test for the significance of association between the independent (predictor) and dependent (outcome) variables in the categorical variables. The level of significance was considered as P < 0.05. Multivariate logistic regression analysis was used to determine predictor variables that predict the outcome.

### Ethical consideration

Ethical approval to conduct the study was sought from the WBUCHS/BMC joint institutional ethic review committee before the commencement of the study.

## Results

One hundred-eighteen cases of tetanus were managed during the period under study. Of these, complete information was available on 102 (86.4%) cases while there was some missing data on 16 (13.6%) cases. Thus, a total of 102 patients were studied with an average of 10 cases per year (range of 8 - 14 cases per year).

### Demographic data

Males were 94 (92.2%) and females were 8 (7.8%) with a male to female ratio of 11.8: 1. Their ages ranged from 8 to 72 years with a mean of 36.21 ± 14.64 years. The median was 34.00 years. The mean age of males and females was 35.14 ± 14.82 and 32.44 ± 11.22 years, respectively (P-value > 0.001). The modal age group was 31-40 years. Seventy-six (74.5%) were below 40 years of age, while 26 (25.5%) were aged 40 years and above. No cases of neonatal tetanus were reported. The majority of patients were farmers (51.0%) (Table 1).

**Table 1:** Distribution of occupation and the portals of entry of tetanus

Variable	Response	Number of patients	Percentage
**Occupation**	Farmers	52	51.0
	Labour/industrial workers	22	21.6
	Civil servant/businessman	6	5.8
	Housewives	5	4.9
	Students	5	4.9
	Unknown	12	11.8
**Portals of entry**	Acute injury (prick, puncture, laceration, burns)	54	52.9
	Skin ulcers	6	5.9
	Local surgical procedures	3	2.9
	• Uvulectomy	1	
	• Circumcision	1	
	• Tooth extraction	1	
	Chronic otitis media	2	1.9
	Others (cellulitis/gangrene)	2	1.9
	Abortion	1	0.9
	No identified portal of entry	34	33.6
**Anatomical site of the portal of entry**	Lower limbs	55	53.8
	Upper limbs	5	4.9
	Head/neck	5	4.9
	Trunk	2	1.9
	Genitalia	1	0.9
	Unknown	34	33.6

### Previous tetanus immunization history

Previous tetanus immunization status was recorded in all patients. Of these, only twenty-four (23.5%) patients had prior tetanus immunization, while the other seventy-eight (76.5%) patients were not vaccinated or did not know their tetanus immunization status. However, in patients who had prior tetanus immunization there was no written proof of the immunization schedule in any cases. Serology test to detect anti-tetanus antibodies was not performed.

### Portals of entry and type of injury

Acute injuries such us prick, puncture, laceration, burns were the most common portals of entry in 52.9% of cases and commonly occurred in the lower limbs (53.8%). The portals of entry were not identified in 33.6% of cases (Table 1). Twenty-one (38.9%) patients had medical wound care before hospital admission but none received tetanus immunoglobulin despite the absence of tetanus immunity.

### Incubation time and period of onset

The incubation period, defined as the time between the inoculation of the wound and the onset of the symptoms, was known in eighty-eight (86.3%) patients. The incubation period ranged from 3 to 26 days with a mean and median of 8.62 ± 4.34 and 7.8 days respectively. The majority of patients, 64 (72.7%) had incubation period of less than 7 days and all of them had severe disease. The period of onset, defined as the interval between the first symptoms and the first spasm, was documented in 65 (63.7%) and ranged from 2 to 9 days with the mean and median of 3.8 ± 2.2 days and 3.2 days respectively.

### Clinical presentation

Ninety-nine (97.1%) patients had generalized tetanus and the remaining two (1.9%) and one (0.9%) patients had cephalic and localized tetanus. respectively. No cases of neonatal tetanus were recorded. Assessment of severity according to Ablett classification system (Table 2) revealed that sixty-six (64.8%) patients had severe disease. Eight (7.8%) and four (3.9%) patients had moderate and very severe disease. Assessment of severity was not recorded in twenty-four (23.5%) patients.

**Table 2:** Ablett Classification of the Severity of Tetanus [16]

Grade	Severity	Clinical features
I	Mild	Mild to moderate trismus; general spasticity; no respiratory embarrassment; no spasms; little or no dysphagia
II	Moderate	Moderate trismus; well-marked rigidity; mild to moderate but short spasms; moderate respiratory embarrassment with an increased respiratory rate > 30, mild dysphagia
III	Severe	Severe trismus; generalized spasticity; reflex prolonged spasms; respiratory rate > 40; apnoeic spells, severe dysphagia; tachycardia > 120
IV	Very severe	Grade III and violent autonomic disturbances involving the cardiovascular system. Severe hypertension and tachycardia alternating with relative hypotension and bradycardia, either of which may be persistent.

Body stiffness/spasm (100%), trismus (100%) and dysphagia (51.25%) were the three commonest presenting complaints (Table 3).

**Table 3:** Clinical presentation of 102 tetanus patients

Clinical presentation	Number of patients	Percentages
Body stiffness/spasm	102	100
Trismus	102	100
Dysphagia	65	63.7
Body aches	24	23.5
Backache	12	11.8
Fever	11	10.8
Headache	9	8.8
Abdominal pain	8	7.8
Jaw pain	4	2.9
Shortness of breath	4	2.9
Urinary retention	2	1.9
Chest pain	2	1.9

### The pattern of tetanus admission

Eighty-four (82.4%) patients were admitted in the ICU for isolation and ventilatory support and the remaining eighteen (17.6%) patients were admitted in isolation rooms in the general wards.

All the patients who were admitted in the ICU required ventilatory support. Mechanical ventilation was used in only 32 (31.4%) cases. The average days on ventilatory support were 16.4 days (1-34 days). Of the patients who were admitted in the wards, 11(61.1%) patients were later transferred to ICU for ventilatory support and close monitoring.

### Treatment of patients

Almost all patients were managed with tetanus toxoid, human tetanus immunoglobulin, antibiotic therapy (penicillin and metranidazole), wound care (wound toilet and debridement), muscle relaxants, sedatives, heparin prophylaxis and artificial ventilation. Tracheostomy was performed in 16 (15.7%) patients and mechanical ventilation was used in 32 (31.4%) cases. Supportive treatment such as balanced fluid and calorie intake, prevention of gastric stress ulcer, prevention of pressure sores were provided in all patients.

### Complications

Complications of tetanus were documented in 56 (54.9%) patients. These included respiratory complications (pneumonia, respiratory failure) in 18 (32.1%) patients, cardiovascular (tachycardia, hypertension) in 11(19.6%), gastrointestinal complications (paralytic ileus) in 10 (17.9%), renal complications (renal failure) in 4 (7.1%), neurological complications (seizures) in 10 (17.9%) and others in 3 (5.4%).

### Outcome of tetanus patients

**Figure 1:**
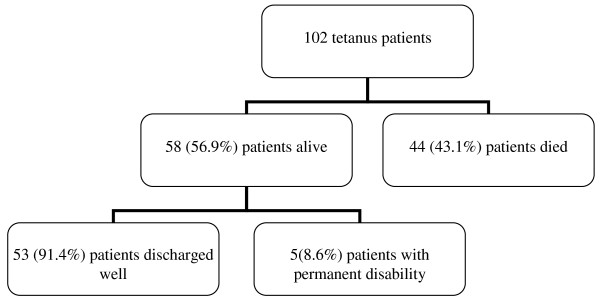
**Flow chart showing the outcomes of the 102 tetanus patients in our study**.

Of the 102patients, 58 (56.9%) patients were alive. of these, 53 (91.4%) patients were discharged well and the remaining 5(8.6%) patients were discharged with permanent disabilities such as persistent vegetative state due to hypoxic brain damage (2 patients), limb amputation (2 patients) and persistent abnormal gait in 1 patient. There were forty-four deaths, accounting for an overall mortality of 43.1% (Figure 1).

Majority of the deaths occurred in the first few days: 11 (25.0%) died in the first 3days while 33 (75.0%) % died in the first 10 days. Of the patients who were discharged alive, only 17 (29.3%) received tetanus toxiod before discharged. The predictors of mortality according to univariate and multivariate logistic regression analyses are shown in table 4.

**Table 4:** The predictors of mortality according to univariate and multivariate logistic regression analyses

Predictor variable	Number of survivors (N/%)	Number of non-survivors (n/%)	Univariate analysis		Multivariate analysis	
			
			O.R. (95% C.I.)	P-value	O.R. (95% C.I.)	P-value
**Age (years)**						
< 40	56 (73.7%)	20 (26.3%)				
≥ 40	2(7.7%)	24 (92.3%)	3.4 (2.8-5.2)	0.012	5.8(3.7-17.3)	**0.002**
**Sex**						
Male	54 (47.4%)	40(42.6%)				
Female	4 (50.0%)	4 (50.0%)	0.2 (0.1-5.4)	0.675	0.4 (0.3-2.1)	0.986
**Incubation period(days) (N = 88)**						
< 7	24 (37.5%)	40(62.5%)				
≥ 7	20(83.3%)	4 (16.7%)	6.3(3.6-9.7)	0.002	0.5(0.3-0.9)	**0.014**
**Period of onset (hours) (N = 65)**						
< 48	11 (29.7%)	26 (70.3%)				
≥ 48	10 (35.7%)	18 (64.3%)	0.4 (0.2-2.6)	0.561	1.7 (0.5-3.2)	0.937
**Prior immunization**						
Yes	16(66.7%)	8 (33.3%)				
No	42(53.8%)	36(46.2%)	3.9 (0.4-6.2	0.068	0.9 (0.5-2.5)	0.561
**Admission pattern**						
ICU	48(57.1%)	36 (42.9%)				
Wards	10 (55.6%)	8 (44.4%)	4.4(0.6-6.7)	0.491	3.8(0.7-4.9)	0.849
**Type of tetanus**						
Generalized	56 (55.6%)	43 (43.4%)				
Cephalic	1(50.0%)	1 (50.0%)	2.5 (0.9-3.1)	0.067	1.7(0.2-5.4)	0.082
Localized	1(100%)	-				
**Severity of tetanus (N = 78)**						
Moderate	7(87.5%)	1 (12.5%)				
Severe	27(38.6%)	43 (61.4%)	2.4(1.3-6.3)	0.012	4.7(2.5-9.1)	**0.001**
**Debridement done**						
Yes	34 (63.0%)	20 (37.0%)				
No	24 (50.0%)	24 (50.0%)	2.4(0.6-3.9)	0.075	5.1(0.9-6.8)	0.089
**Tracheostomy done**						
Yes	14 (87.5%)	2 (12.5%)				
No	44(51.2%)	42(48.8%)	3.1(1.4-7.3)	0.011	4.9(2.3-8.1)	**0.004**
**Need for ventilatory support**						
Yes	26(81.3%)	6(18.7%)				
No	32 (45.7%)	38 (54.3%)	1.7(1.1-4.5)	0.032	0.2 (0.1-0.8)	**0.013**
**Complications**						
Present	35 (62.5%)	21 (37.5%)				
Absent	23(50.0%)	23 (50.0%	3,9(0.5-4.3)	0.063	1.6(0.4-6.2)	0.911

Average ICU stay was 19.3 days (range 1-26 days) and the overall mean duration of hospital stay was 34.12 ± 38.44 days (1-120 days). The median duration of hospitalization was 32.00 days. The mean and median duration of hospitalization for non-survivors were 6.2 ± 4.8 days (1-28 days) and 5.8 days respectively.

## Discussion

Tetanus is still prevalent in developing countries and constitutes significantly to high morbidity and mortality despite the documented effectiveness of tetanus vaccines and its availability since 1923 [1-3]. High incidence of tetanus admissions in developing countries including Tanzania is attributed to low levels of health awareness in terms of vaccination and availability of human and material resources to manage the disease [4,7]. This observation is reflected in our study as more than three quarters of our patients were not vaccinated or did not know their tetanus immunization status. This finding calls for preventive measures to reduce the incidence of this disease, such as wide immunization coverage and health education.

In agreement with other studies in developing countries [4,13,14,16], tetanus patients in the present study were quite young which is in contrast to other studies in developed countries [8,9]. This observation can be explained by the fact that in developing countries tetanus is common in the young due to lack of effective immunization program and inappropriate treatment of injuries [4,7] whereas in developed countries tetanus occurs mainly in elderly due to decline in protective antibodies [5,6].

In this study, male patients were more affected than females. The male preponderance in this study has been reported elsewhere [4,6,8,9,11,12]. This could be explained by the fact that men tend to spend more time outdoor, in farming activities and other types of fieldwork. Hence, they are more likely to be exposed to both the causal organism, *C. tetani*, which is ubiquitous in soil in a tropical country like Tanzania and the penetrating injury necessary for the organism to enter the body. The high proportion of admission among males in this study also reflects the low vaccination rates among males in the community as compared to females and children who gets their vaccination during pregnancy and childhood respectively. Health education on the importance of vaccination among males is highly needed to prevent from contracting this serious disease.

Majority (51.0%) of the tetanus patients in this study were farmers which is in agreement with other studies [6,8]. This observation is in contrast to a Nigerian study which reported students and civil servants as the majority of cases [16]. This pattern of occupational risk group in our study can be explained by the fact that farmers or the peoples who live in the rural areas and engage themselves in the agricultural sector are more likely to be exposed to the causal organism as well as the injury necessary for the organism to enter the body.

In agreement with other studies [8,9,16,17], the most common portal of entry in this study was injuries in the lower limbs. This is in contrast to Joshi *et al *[12] who reported upper limbs as the most common portal of entry. This lower limb preponderance in this study could be explained by the fact that C. *tetani *exists in soil; hence, any lower limb injury would be open to contamination and infection by this organism, bearing in mind too that most tetanus patients were rural farming folks. Also, the preponderance of lower limbs in our study is thought to result from poor protective footwear. The portals of entry were not identified in 33.6% of cases reflecting that the injuries were likely to be trivial to be recalled. Trivial wounds on the lower limbs as possible portals of entry for tetanus infection are common because most people in the rural areas do not wear shoes.

Body stiffness/spasm, trismus and dysphagia, in that order, were the commonest complaints of the tetanus patients in our series which is in agreement with other studies [8,9,11,14]. Hence, a high index of suspicion for tetanus is of paramount whenever patients present with any of these symptoms as tetanus is essentially a clinical diagnosis and laboratory results as well as cultures are of little diagnostic value [5]. If a patient presents with all the three complaints, the probability of tetanus would be extremely high.

The treatment of tetanus patients requires a well established intensive care facility with a medical and nursing staff experienced in treating artificially ventilated and haemodynamically unstable patients. The majority (82.4%) of study patients required ICU management an observation which is also reported in other studies [9,11]. However, ICU admission in this study did not significantly improve the prognosis of these patients in terms of mortality. This may be attributed to low levels of tracheostomy and mechanical ventilation which were performed in only 15.7% and 31.4% of cases respectively.

In this study, tracheostomy to circumvent the problem of laryngeal spasm (which could lead to asphyxiation and hypoxia) and to enable tracheal suction and toilet to be carried out efficiently (airway protection) was performed in only 15.7% of patients which is similar to what was reported by Feroz and Rahman in Bangladesh [8]. This is in contrast with other studies which reported that all tetanus patients underwent tracheostomy [9,18]. The use of tracheostomy in the management of patients with severe tetanus will undoubtedly prevent death due to asphyxia from laryngeal muscle spasm (and acute airway obstruction), respiratory muscle spasm and aspiration [18]. The low rate of tracheostomy in our study may be responsible for high mortality rate among tetanus patients. There was no obvious explanation for the low rate of tracheostomy in this study.

Complication rate in the present study is high compared to other studies [6,11]. However, the presence of complication did not significantly affect the outcome of tetanus patients. Our complication pattern was fairly similar to what was reported by Feroz and Rahman in Bangladesh [8]. We could not find any obvious reason in literature to explain this similarity. Much attention must therefore be paid to prevent these complications through early diagnosis and management.

The prognosis of patients with tetanus has been reported variably. Overall, mortality is approximately 10-50%, however in certain age groups e.g. neonates it is as high as 90-95% [19]. In this study, mortality rate was 43.1% which is comparable with the observation reported by Mohammed *et al *[20], whereas Mchembe & Mwafongo [4] in Tanzania and Zziwa [21] in Uganda have reported higher mortality rate of 72.7% and 47% respectively. The high mortality rate could be due to the gross inadequacy of human and material resources to manage severe tetanus in the intensive care unit, typical of developing countries like Tanzania [4,22]. Various factors have been known to affect the prognosis [11]. The poor prognostic factors in this study included age ≥ 40 years, shorter incubation periods (< 7 days), low rate of tracheostomy, and severity of tetanus. Most of the deaths in our series were attributed to sudden cardiac arrest, respiratory failure and infective pulmonary complications, an observation similar to other studies [8,21]. In this study, only 29.3% of the patients who were discharged cured received tetanus toxiod before discharged a figure fairly consistent with that of other studies [21,22]. This finding calls for a need to provide health education on primary immunization and scheduled booster immunization that have greatly found to reduce the incidence of tetanus.

The overall mean duration of hospital stay in this study was 34.12 ± 38.44 days (1-120 days) which is high compared to other studies [4,9,12,16,17]. In one study, the overall mean duration of hospital stay was 83.0 days [8]. Prolonged duration of hospital stay has an impact on hospital resources as well as on increased cost of heath care, loss of productivity and reduced quality of life.

The potential limitation of this study is the fact that information about some patients was incomplete in view of the retrospective nature of the study. This might have introduced some bias in our findings.

## Conclusion

Although tetanus is a vaccine preventable infectious disease, its prevalence is still high in our environment and it remains a difficult to treat disease with an unacceptably high morbidity and mortality rate, even with available advanced facilities for its management. Young adult males are commonly affected. The incidence of tetanus can be reduced significantly by an effective immunization program and proper wound management of the patients. Early recognition, intense support and prompt treatment improves morbidity and mortality of patients diagnosed with tetanus. Our study show comparable clinical pattern and outcome with other studies in the developing countries reported in the literatures.

## Competing interests

The authors declare that they have no competing interests.

## Authors' contributions

PLC designed the study, contributed in literature search, data analysis, manuscript writing & editing and submission of the manuscript. JBM, RMD, NM and SM participated in study design, data analysis, manuscript writing and editing JMG participated in study design, supervised the write up of the manuscript and edited the manuscript before submission. All the authors read and approved the final manuscript.

## References

[B1] Galazka A Gasse F The present status of tetanus and tetanus vaccination Curr Top Microbial Immunol 1995 195 31 53 10.1007/978-3-642-85173-5_2 8542758

[B2] Anuradha S Tetanus in adults-A continuing problem: An analysis of 217 patients over 3 years from Delhi, India, with special emphasis on predictors of mortality Med J Malaysia 2006 61 1 7 14 16708728

[B3] Oladiran I Meier DE Ojelade AA Olaolorun DA Adeniran A Tarpley JL Tetanus continuing problem in the developing world World J Surg 2002 26 10 1282 85 10.1007/s00268-002-6497-z 12209228

[B4] Mchembe MD Mwafongo V Tetanus and its treatment outcome in Dar es Salaam: need for male vaccination East African Journal of Public Health 2005 2 22 23

[B5] Sandford JP Tetanus-Forgotten but not gone N Engl J Med 1995 332 812 3 10.1056/NEJM199503233321209 7862186

[B6] Amare A1 Yami A Case-fatality of adult Tetanus at Jimma University Teaching Hospital, Southwest Ethiopia African Health Sciences 2011 11 1 36 40 21572855 PMC3092314

[B7] Dietz V Milstien JB van Loon F Cochi S Bennett J Performance and potency of tetanus toxoid: implications for eliminating neonatal tetanus Bull WHO 1996 74 619 28 9060223 PMC2486793

[B8] Feroz AHM Rahman MH A Ten-year Retrospective Study of Tetanus at a Teaching hospital in Bangladesh J Bangladesh Coll Phys Surg 2007 25 62 69

[B9] Lau LG Kong KO Chew PH A ten-year retrospective study of tetanus at a general hospital in Malaysia Singapore Med J 2001 42 8 346 50 11764050

[B10] Edlich RF Hill LG Mahler CA Cox MJ Becker DG Horowitz JH Management and prevention of tetanus J Long Term Eff Med Implants 2003 13 3 139 54 10.1615/JLongTermEffMedImplants.v13.i3.20 14516181

[B11] Younas NJ Abro AH Das K Abdou AMS Ustadi AM Afzal S Tetanus: Presentation and outcome in adults Pak J Med Sci 2009 25 5 760 765

[B12] Joshi S Agarwal B Malla G Karmacharya B Complete elimination of tetanus is still elusive in developing countries: a review of adult tetanus cases from referral hospital in Eastern Nepal Kathmandu Univ Med J (KUMJ) 2007 5 3 378 81 18604058

[B13] Adekanle O Ayodeji OO Olatunde LO Tetanus in a Rural Setting of South-Western Nigeria: a Ten-Year Retrospective Study Libyan J Med 2009 4 100 4 10.4176/081125 PMC3066715 21483514

[B14] Komolafe MA Komolafe EO Ogundare AO Pattern and outcome of adult tetanus in lle-lfe, Nigeria Niger J Clin Prac 2007 10 4 300 303 18293639

[B15] Ablett JJL Ellis M Analysis and main experience in 82 patients treated in Leeds tetanus unit 1967 Symposium on tetanus in Great Britain. Leeds 1

[B16] Chukwubike OA God'spower AE A 10-year review of outcome of management of tetanus in adults at a Nigerian tertiary hospital Ann Afr Med 2009 8 3 168 172 19884693 10.4103/1596-3519.57239

[B17] Fawibe AE The Pattern and Outcome of Adult Tetanus at a Sub-urban Tertiary Hospital in Nigeria Journal of the College of Physicians and Surgeons Pakistan 2010 20 1 68 70 20141700

[B18] Fasunla AJ Challenges of Tracheostomy in Patients Managed for Severe Tetanus in a Developing Country Int J Prev Med 2010 1 3 176 181 21566788 PMC3075528

[B19] Bhatia R Parbharkar S Grover VK Tetanus Neurol India 2002 50 398 407 12577086

[B20] Mohammed W Bhojo AK Nashaa T Rohma S Nadir AS Aseem S Autonomic nervous system dysfunction predicts poor prognosis in patients with mild to moderate tetanus BMC Neurology 2005 5 2 10.1186/1471-2377-5-2 15679900 PMC548694

[B21] Zziwa GB Review of tetanus admissions to a rural Ugandan Hospital 2009 7 3 UMU press 199 202

[B22] Aboud S Budha S Othman MA Tetanus at Mnazi Mmoja Hospital in Zanzibar, Tanzania TMJ 2001 16 3 5 7

